# Predictors of Maternal Depression Management among Primary Care Physicians

**DOI:** 10.1155/2010/671279

**Published:** 2010-03-25

**Authors:** Jenn A. Leiferman, Sarah E. Dauber, Katie Scott, Kurt Heisler, James F. Paulson

**Affiliations:** ^1^Colorado School of Public Health, 13001 E 17th Place, Mailstop B119, Aurora, CO 80045, USA; ^2^Columbia University, USA; ^3^Colorado State University, USA; ^4^Eastern Virginia Medical School, USA

## Abstract

*Purpose*. The present surveillance study examined predictors of the management of maternal depression in primary care settings. *Methods*. A total of 217 physicians completed a 60-item survey assessing demographics, physicians' attitudes, beliefs, efficacy, current practices, and perceived barriers regarding the management of maternal depression. Structural equation modeling was used to estimate a model that examined the relationships among physicians' knowledge, beliefs, self-efficacy, perceived barriers, past training toward and current management practices for maternal depression. *Results*. In a model predicting physician depression management practices, a good overall fit was observed (*χ* ^2^ = *136.63*, CFI = .97, TLI = .95, RMSEA = .05), with physician comfort with, confidence in, and perceived responsibility for managing maternal depression all having prominent positive associations. *Conclusions*. These findings will guide the development of future multifaceted intervention strategies to enhance physician skills in managing maternal depression in primary care settings.

## 1. Background

Depression is common among women, particularly during their child-bearing years with prevalence rates ranging from 10%–20% [1]. Maternal depression not only negatively impacts the health of the mother but often directly or indirectly influences her children's well-being resulting in poorer cognitive, social, and emotional child outcomes [2–6]. Depression which alters parenting behavior is one proposed pathway in which depression indirectly impacts child wellbeing. Compared to their nondepressed counterparts, depressed mothers engage in fewer behaviors that have a positive impact on child health and more parenting behaviors that result in poorer child outcomes [7–9]. These detrimental effects appear to extend beyond early childhood such that older children of depressed mothers experience depression, substance abuse, and conduct disorders at rates higher than children of nondepressed mothers [10–14]. 

Although the adverse consequences of maternal depression have been well-established, maternal depression often goes undetected in primary care settings. For instance, pediatricians in urban settings often miss cases of maternal depression [15, 16]. Moreover, if screening for maternal depression is conducted in a primary care setting, such as in an obstetric clinic, it is often done infrequently [17]. 

Mothers make frequent visits to primary care providers (PCPs) such as obstetricians, pediatricians, and family medicine physicians, which makes the disconnect between the prevalence of maternal depression and its inadequate management in primary care settings all the more troublesome. These visits are often mothers' sole contact with the health care system, and provide an opportunity to manage maternal depression [18, 19]. Further, mothers also report being receptive to receiving mental health care in these primary care settings [16, 19]. Despite these factors, managing maternal depression in many primary care settings does not currently occur and there has been little research to identify why this is so [20, 21]. There is a definite need to better understand the role of barriers and facilitators such as those related to physicians' education, attitudes, beliefs, efficacy, practices, and systematic factors, that contribute to this disconnect. Moreover, research examining these barriers has lacked a comprehensive approach and has been done mostly in the context of the management of depression in the general population [22, 23]. The present study stems from previous work which examined differences in attitudes, beliefs, efficacy and barriers related to managing maternal depression among three types of PCPs (i.e., obstetricians, pediatricians, and family medicine practitioners) and found significant differences in beliefs, perceived barriers, and practices across specialties [20]. Moreover, approximately 40% of the PCPs reported rarely or never managing maternal depression in their practices, underscoring the need to better understand ways to enhance the management of maternal depression in primary care settings [20]. 

**Figure 1:**
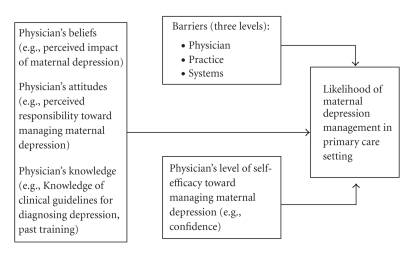
Conceptual model for the examination of factors related to management of maternal depression.

The present study extends this work by using a comprehensive approach and theory-driven conceptual model to examine the interplay of the aforementioned potential barriers and facilitators to the management of maternal depression in primary care settings (Figure 1). This model stems from the Health Belief Model and the Social Ecological Model, such that in accordance with the Health Belief Model, we examined physicians' perceived beliefs concerning the potential impact of maternal depression, and assessed their attitudes, barriers, knowledge, and self-efficacy toward the management of maternal depression [24]. In line with the Social Ecological Model, we hypothesized that the barriers a physician experiences are nested factors which exist at the individual, practice and system levels and each barrier may be influenced by a larger, encompassing barrier surrounding it [25]. Based on this conceptual model we hypothesized (1) that physicians' beliefs, attitudes, self-efficacy, and knowledge would predict their management of maternal depression, and (2) that particular barriers may impede the likelihood of this behavior.

## 2. Methods and Materials

### 2.1. Sample

The sample comprised 217 PCPs practicing medicine in one of three specialties (i.e., family medicine, obstetrics, and pediatrics) in Southeastern Virginia. The sample included 87 family medicine physicians (40.1%), 81 pediatricians (37.3%), and 49 obstetricians (22.6%). Demographics for the full sample and by specialty are located in Table 1.

### 2.2. Procedures

Prior to conducting the present study, the study protocol was approved by the relevant Institutional Review Board. Eligible PCPs included physicians currently practicing in one of three specialties: obstetrics, pediatrics, or family medicine and in one of the five designated cities in the Hampton Roads Area in Southeastern Virginia. Eligible PCPs who met the study inclusion criteria were identified through the Virginia Board of Medicine website, local hospital, and chapter directories. The medical executive of the local hospital sent eligible participants a pre-notification email to inform them about the upcoming study. Approximately one week later the PI sent eligible participants an email and/or facsimile with a cover letter containing the link to the web survey. All eligible participants received up to 4 follow-up notifications either by facsimile, email, or postal mail which gave them the opportunity to complete the survey by mail or web. A total of 232 completed surveys were returned out of 971 PCPs in the initial pool. Seventy nine people responded by mail and the remaining 153 completed the survey online. This represents a response rate of 23.9%. Detailed information regarding sampling procedures can be found in Leiferman et al., 2008 [20].

### 2.3. Measures

Based on the conceptual model in Figure 1, an online survey was developed to assess PCPs' attitudes, beliefs, barriers, and practices regarding the assessment and treatment of maternal depression (for more details related to survey development please see [20]). The final survey consisted of 60 items and took approximately 15 minutes to complete. Demographics were assessed at both the physician level (e.g., race, years of practice) and at the practice level (e.g., location and type of practice). PCPs were also asked to rate the extent of their agreement with a series of statements regarding attitudes, beliefs, knowledge and efficacy toward maternal depression on a six-point Likert-type scale (e.g., strongly agree-strongly disagree). PCPs were also asked to describe their current management of depression practices; perceived barriers toward the management of maternal depression in their practice at the patient, physician, and system levels; and previous training related to maternal depression.

### 2.4. Data Analyses

Structural Equation Modeling (SEM) was used to estimate the effects of physicians' beliefs, attitudes, knowledge, perceived barriers, and self-efficacy on the likelihood of maternal depression management in primary care settings as per the conceptual model presented in Figure 1. This method is particularly useful here because it allows for the simultaneous examination of direct effects (as in multivariable regression), creation of latent or measured variables (as in confirmatory factor analysis), and the estimation of indirect or mediated effects. Within this SEM framework, the variables were treated as continuous and tests of indirect effects were also performed to evaluate the mediating roles hypothesized in the conceptual model. Indirect effects, in the context of SEM, can provide a single-model test of mediation, provided the proper study design elements are taken into consideration, but in brief reflect the amount of influence that a presumably influential variable has on an outcome via and intermediate variable (the reader is referred to Baron and Kenny's seminal work for more information on this topic) [26]. In this study, evidence of mediation is interpreted more cautiously, as the data are cross-sectional and limit causal assertions. Full Information Maximum Likelihood estimation was used to fit all SEMs, thereby allowing model estimation with all available data. Model fit was assessed using the Tucker-Lewis Non-normed Fit Index (TLI), the comparative fit index (CFI) and root mean square error of approximation (RMSEA) using guidelines proposed by Hu and Bentler [27].

**Confirmatory Factor Analysis and Exploratory Factor Analysis**

Since using Confirmatory Factor Analysis resulted in poor fit according to the conceptual model, the items were entered into an Exploratory Factor Analysis (EFA). The initial EFA was used to cull items with poor or ambiguous loadings. All factors were entered into initial models and dropped in a second round model if they proved to be non-significant. Unless otherwise stated, “significant” effects are associated with test statistics with *P* < .01 unless otherwise stated. Univariate modeling was carried out using Stata version 8.1 software (Stata Corporation, 2004). All SEM analyses used Mplus version 4.1 [28].

**Modeling**

Modeling in the present study included latent variables that were measured by several indicators:

*Management practices* were measured by assessment, screening, treatment, and referral (e.g., How often do you assess for maternal depression?; How often do you use a screening tool to help in your diagnosis of maternal depression?; How often do you refer a patient for treatment of maternal depression?). Thus, management practices were measured individually and each contributed to the latent variable that synthesized management practices into one factor.

**Figure 2:**
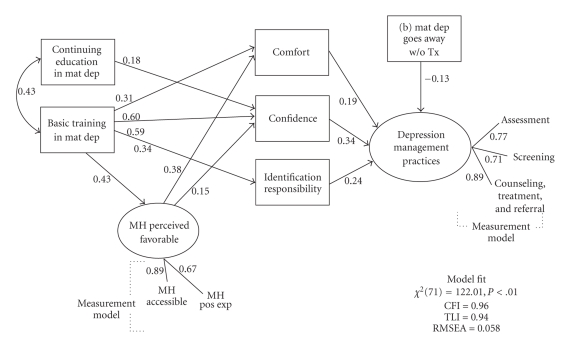
Fitted structural equation model predicting physician depression management practices. note: All paths shown are standardized and have associated *P* < .01. Paths with associated *P* < .01 are noted or are not shown. A latent variable *Percieved Negative Patient Bieliefs *is modeled, but not displayed here for clarity. MH: Mental Health, Mat Dep: Maternal Depression, Tx: Treatment, *Based on sample (*N* = 217).

*Attitudes* were assessed by the perceived level of responsibility for identification and follow-up care, as well as perception of current mental health services. *Identification responsibility* reflected responses to statements such as “Recognizing maternal depression is my responsibility”. *Follow-Up Responsibility* was measured by perceived responsibility and time for followup (e.g., It is my responsibility to follow-up after making a referral to a mental health specialist; I do not have time to follow up with the patient after making a referral). *Mental Health Perceived Favorable *attitudes were measured by perceived accessibility of mental health services and positive experiences with mental health (e.g., I am very satisfied with my access to mental health professionals in my community. I am satisfied with my experiences with consulting with mental health professionals).* Self-efficacy* was assessed by perceived level of comfort and confidence. For example, *perceived level of comfort* was assessed by Likert-type responses to statements such as “I feel comfortable talking about depression with patients.” and “ I am comfortable contacting a mental health professional to consult about a patient.” *Perceived confidence* was assessed by Likert-type responses to statements such as “ I feel confident in my ability to diagnose maternal depression”.* Knowledge* including *Basic training in maternal depression *and *continuing education in maternal depression*, was also assessed directly by Likert-type items. Basic training was measured by items such as “How would you rate your profession training in how to diagnose maternal depression? How familiar are you with DSM-IV criteria for diagnosing depression? Continuing education was measured by items such as “Have you ever received continuing education training related to postpartum depression?” and “Have you ever received continuing education training related to depression during pregnancy?”* Beliefs* were assessed by responses to the question “Maternal depression will go away without treatment”.* Barriers* were assessed at multiple levels: physician (e.g., perceived patient barriers such as patient is in denial, believe depression is normal), practice and system barriers (e.g., lack of in-house mental health specialist, limited time, inadequate mental health resources, financial barriers). After initial model fit was estimated, several iterations were used to refine the model by dropping variables that were not statistically significant or which detracted from overall model fit, with an inclusion threshold of *P* < .10. Model modifications are described below and the final model that is displayed in Figure 2 includes values with associated *P*-values below  .01 (with the exception of “Maternal depression goes away without treatment,” *P* = .02).

## 3. Results

After initial model fitting, several elements of the conceptual model were dropped due to a lack of statistical significance. These included all items measuring physician beliefs except for “Maternal depression goes away without treatment” and beliefs that patients were stigmatized by or would not respond well to attempts to assess and manage maternal depression;” as well as all items measuring Physician (e.g., perceived patient barriers such as patient is in denial, believe depression is normal), Practice and System barriers (e.g., lack of in-house mental health specialist, limited time, inadequate mental health resources, financial barriers). 

A final model that included only variables with *P* < .10 demonstrated good overall fit (*χ* ^2^[71] = 122.006, Comparative Fit Index [CFI] = .959, Tucker-Lewis Non-normed Fit Index [TLI] = .941 root mean square error of approximation [RMSEA] = .058). Physician attitudes, self-efficacy, knowledge, and training all played prominent roles in predicting depression management practices (Figure 2). In addition, several indirect (mediated) effects were observed. The first was from Continuing Education to Management Practices via Confidence, such that physicians who had more continuing education coursework on the topic were more likely to actively manage maternal depression because of higher self-reported confidence (standardized indirect effect = .061, *z* = 2.378, *P* = .017). Second, physicians who reported more favorable perceptions of mental health services were more likely to actively manage maternal depression because of associated increased confidence (standardized indirect effect = .051, *z* = 2.086, *P* = .037) and comfort (standardized indirect effect = .073, *z* = 2.581, *P* = .010). Third, physicians who reported better training and higher levels of knowledge were more likely to actively manage maternal depression because they were more confident (standardized indirect effect = .204, *z* = 3.202, *P* = .001), comfortable (standardized indirect effect = .060, *z* = 2.494, *P* = .013), and felt greater responsibility (standardized indirect effect = .139, *z* = 2.597, *P* = .009).

## 4. Discussion

There is a growing body of evidence suggesting the importance of the management of maternal depression in primary care practices [15–17, 20]. In order to inform future intervention development, the present study examined potential determinants of management of maternal depression practices in primary care settings. As previously published, nearly 40% of the PCPs in this sample reported never or rarely assessing for maternal depression, less than 30% reported current use of a screening tool for maternal depression, and approximately 60% reported rarely or never providing counseling or referring patients who are depressed for follow-up mental health care [20]. Clearly there is a need to better understand the determinants of maternal depression management practices to inform future intervening efforts.

Based on our conceptual model (see Figure 1), we proposed that PCPs' beliefs, attitudes, self-efficacy, and knowledge would predict management of maternal depression and that particular barriers may impede the likelihood of this behavior as well. Knowledge via past training (i.e., basic training or CME) for managing maternal depression seems to play an integral role in whether a physician will engage in maternal depression management practices. Our findings suggest that physicians who reported better training and higher levels of knowledge were more likely to report actively managing maternal depression in their practices. Unfortunately, over 60% of the PCPs reported that their past training in diagnosis or treatment of maternal depression was either fair, poor, or never received [20]. These findings, coupled with past research suggesting that mental health training is limited within residency training, and that physicians often report that they do not feel they have had adequate training to correctly manage depression highlight the need for more education and training of primary care practitioners in the area of maternal depression [22, 29]. 

In addition to knowledge as a predictor, our findings suggest that self-efficacy and attitudes mediate the relationship between knowledge/past training and management practices. Self-efficacy, assessed in this study by level of perceived confidence and comfort in managing maternal depression, is often cited as a strong behavioral change agent closely tied to health behavior outcomes [30, 31]. Our study also found PCPs' perceived self-efficacy and attitude (i.e., feeling responsible for identifying maternal depression) as strong predictors of maternal depression management. Conversely, a lack of perceived confidence and a sense of feeling responsible to manage depression have been cited as barriers to managing depression in primary care practices [23, 29]. Not surprisingly, self-efficacy and attitudes related to feeling responsible for identifying women at risk for depression were strongly influenced by knowledge in the form of basic training and to some extent by specialized continuing education.

Attitudes related to the positive perception of mental health services were influenced by knowledge/past training and also linked to self-efficacy such that physicians who reported higher self-efficacy also perceived available mental health services more favorably. This is of particular relevance since past research has reported provider dissatisfaction with current mental health management practices as a systemic barrier toward managing depression in primary care settings [22]. This reinforces the importance of increasing knowledge via basic training and CEU attainment focusing on maternal depression. Experts in implementation research suggest that this training should extend beyond standard education and skill-based training and include ongoing consultation and coaching [32]. Providing ongoing consultation and coaching is one of six integral components (i.e., staff selection, preservice and inservice training, staff and program evaluation, facilitative administrative support, and systems interventions) in implementing successful change within organizations [32]; in this case, change in PCPs depression management in primary care practices.

Contrary to what we hypothesized, physician beliefs pertaining to perceived impact of maternal depression were not a strong predictor in the model. This outcome is most likely due to the lack of variance in response, as more than 95% of the respondents believed maternal depression to be detrimental. However, the belief that maternal depression goes away without treatment was negatively correlated with management practices.

Surprisingly, the present study did not find often cited barriers related to limited time and financial issues to be determinants of maternal depression management practices. In terms of financial barriers, only 10% of the sample endorsed this item whereas it was more of an even split related to time. For both factors, measurement most likely impacted our findings as both potential factors were measured by dichotomous (yes/no) items instead of a Likert-type measurement. The other possibility may be that other elements in the model, like perceived responsibility, washed out any influence that time or financial considerations might have had. Financial issues have been suggested to play a role as capitation rates often exclude mental health services and few incentives exist for mental health specialists to collaborate with primary care providers [33]. However, contrary to what we expected, barriers related to financial issues were not strong predictors of maternal depression management practices. 

### 4.1. Limitations

Findings from our study should be evaluated in light of the following limitations. Despite using techniques linked to optimal response rates [34], our response rate was suboptimal (see the work of Leiferman et al. (2008) [20] for more on sampling and nonresponse limitations). Findings from this study have limited generalizability as they are based on a small, geographical sample and thus, may not represent other populations. Moreover, this was the first model to be tested in this population, thus may further limit the generalizability of the model; although only relatively strong effects were reported here. Given the small sample size we were not able to conduct models examining differences across the three specialties (i.e., obstetrics, pediatrics and family medicine), thus, more research is warranted in this area.

### 4.2. Implications

Modeling efforts from this study suggest that much of PCPs' practices in identifying and treating maternal depression, when taken together, are tightly linked to attitudes related to feelings of responsibility for those patient issues and self-efficacy/confidence with maternal depression management. Basic training and continuing education in maternal depression seem to be integral in this process as they have a direct impact on management practices, but also indirectly affect management practices through perceived self-efficacy and responsibility. 

Overall PCPs reported that they are open to making modifications to their practice and improving their knowledge and skills related to managing maternal depression. [20] Implementing screening protocols is one potentially effective way to identify maternal depression and initiate prompt treatment of the disorder. A recent study found screening for maternal depression during well child visits to be feasible [35]. Improved screening coupled with strong coordination with mental health services would represent a significant advance in reducing maternal and child health risks and is likely to improve health outcomes. In addition to implementing screening tools, innovative and novel models need to be developed and revised to promote the management of maternal depression. The findings from our previous study suggest that PCPs in general would like more training on mental health topics in the form of CEUs, guidelines, and computer deliverables [20]. In particular, the majority of the PCPs stated they would like information to enhance patient communication about mental health issues. In conclusion, the present study's findings may ultimately help inform the design of future intervention models aimed at improving the management of maternal depression in primary care practices.

## Figures and Tables

**Table 1:** Sample demographics by specialty.

	Full sample (*N* = 217)	Family medicine (*N* = 87)	OB/GYN (*N* = 49)	Pediatricians (*N* = 81)
Gender				
Male	97 (44.7%)	45 (51.7%)	17 (34.7%)	35 (43.2%)
Female	120 (55.3%)	42 (48.3%)	32 (65.3%)	46 (56.8%)
Race				
White	155 (72.4%)	59 (68.6%)	37 (77.1%)	59 (73.8%)
African American	26 (12.1%)	10 (11.6%)	8 (16.7%)	8 (10.0%)
Asian	24 (11.2%)	12 (14.0%)	2 (4.2%)	10 (12.5%)
Other	9 (4.2%)	5 (5.8%)	1 (2.1%)	3 (3.8%)
Years providing healthcare services				
Less than 2	10 (4.7%)	5 (5.7%)	3 (6.4%)	2 (2.5%)
2–5 years	34 (15.8%)	14 (16.1%)	8 (17.0%)	12 (14.8%)
6–10 years	43 (20.0%)	15 (17.2%)	10 (21.3%)	18 (22.2%)
11–15 years	33 (15.3%)	14 (16.1%)	6 (12.8%)	13 (16.0%)
16+ years	95 (44.2%)	39 (44.8%)	20 (42.6%)	36 (44.4%)
Practice setting				
Urban	110 (51.6%)	40 (46.5%)	25 (52.1%)	45 (57.0%)
Suburban	95 (44.6%)	42 (48.8%)	21 (43.8%)	32 (40.5%)
Rural	8 (3.8%)	4 (4.7%)	2 (4.2%)	2 (2.5%)
Years at present location				
Less than 1 year	26 (12.1%)	9 (10.6%)	7 (14.3%)	10 (12.5%)
2-3 years	53 (24.8%)	16 (18.8%)	14 (28.6%)	23 (28.8%)
4–10 years	62 (29.0%)	31 (36.5%)	12 (24.5%)	19 (23.8%)
11–15 years	31 (14.5%)	15 (17.6%)	6 (12.2%)	10 (12.5%)
16+ years	42 (19.6%)	14 (16.5%)	10 (20.4%)	18 (22.5%)

Note. There were no significant differences across specialties.
